# Preliminary Neurophysiological Evidence of Altered Cortical Activity and Connectivity With Neurologic Music Therapy in Parkinson's Disease

**DOI:** 10.3389/fnins.2019.00105

**Published:** 2019-02-19

**Authors:** Isabelle Buard, William B. Dewispelaere, Michael Thaut, Benzi M. Kluger

**Affiliations:** ^1^Department of Neurology, University of Colorado Denver, Denver, CO, United States; ^2^Medical School Program, University of Colorado Denver, Denver, CO, United States; ^3^Department of Music, University of Toronto, Toronto, ON, Canada

**Keywords:** auditory-motor, entrainment, therapy, fine motor, rehabilitation

## Abstract

Neurologic Music Therapy (NMT) is a novel impairment-focused behavioral intervention system whose techniques are based on the clinical neuroscience of music perception, cognition, and production. Auditory Stimulation (RAS) is one of the NMT techniques, which aims to develop and maintain a physiological rhythmic motor activity through rhythmic auditory cues. In a series of breakthrough studies beginning in the mid-nineties, we discovered that RAS durably improves gait velocity, stride length, and cadence in Parkinson's disease (PD). No study to date reports the neurophysiological evidence of auditory-motor frequency entrainment after a NMT intervention in the Parkinson's community. We hypothesized that NMT-related motor improvements in PD are due to entrainment-related coupling between auditory and motor activity resulting from an increased functional communication between the auditory and the motor cortices. Spectral analysis in the primary motor and auditory cortices during a cued finger tapping task showed a simultaneous increase in evoked power in the beta-range along with an increased functional connectivity after a course of NMT in a small sample of three older adults with PD. This case study provides preliminary evidence that NMT-based motor rehabilitation may enhance cortical activation in the auditory and motor areas in a synergic manner. With a lack of both control subjects and control conditions, this neuroimaging case-proof of concept series of visible changes suggests potential mechanisms and offers further education on the clinical applications of musical interventions for motor impairments.

## Background

Neurologic Music Therapy (NMT) is a novel impairment-focused behavioral intervention system whose techniques are based on the clinical neuroscience of music perception, cognition, and production (Thaut, 2014). One of the perceptual and neural mechanisms underlying NMT applications is “rhythmic entrainment” where one system's motion or signal frequency entrains the frequency of another system. In the brain, firing rates of auditory neurons, triggered by auditory rhythms and music, entrain the firing patterns of motor neurons, thus driving the motor system into different frequency levels (Thaut, 2015). Rhythmic Auditory Stimulation (RAS) is one of the NMT techniques, which aims to develop and maintain a physiological rhythmic motor activity through rhythmic auditory cues. Psychophysics studies show auditory cues function as a timekeeper entraining the motor response into a very rapid and temporally precise state of synchronization to the rhythmic cue frequency (Thaut et al., 1999). In cortical sensory areas, auditory-evoked oscillatory rhythms in the beta (15–30 Hz) and gamma (40–80 Hz) frequency range are direct measures of rhythm perception and possibly reflect auditory-motor interactions (Snyder and Large, 2005; Fujioka et al., 2009). Therefore, they are useful to investigating the entrainment-related coupling between auditory and motor activity. Beta oscillations have been holding a crucial role in directional auditory-to-motor coupling during piano playing of non-PD professional pianists (Jäncke, 2012).

Parkinson's disease (PD) is a neurodegenerative illness defined by characteristic motor symptoms including slow and small movements as well as difficulty with movement initiation and disruptions in timing. Several explanations for the underlying pathophysiology include beta and gamma impairments in subcortical structures such as the basal ganglia (BG) (Doyle et al., 2005) as well as in the cortical motor areas (Heinrichs-Graham et al., 2014; Stegemöller et al., 2016). Deep brain stimulation or dopamine replacement therapy restore normal BG beta oscillations (Jenkinson and Brown, 2011) as well as cortical motor networks dynamic (Michely et al., 2015). This suggests that interventions targeting motor symptoms have the ability to influence oscillatory rhythms in the brain or vice-versa.

In a series of breakthrough studies beginning in the mid-nineties we have discovered that auditory rhythmic cues durably improve gait velocity, stride length, and cadence in PD (Thaut et al., 1996; McIntosh et al., 1997). RAS is now recognized as state of the art for mobility treatment for PD (Hove and Keller, 2015), and may occur via a shift from basal ganglia-thalamocortical to other pathways involving possibly the cerebellum (Cunnington et al., 2001; Debaere et al., 2003) or through an effective cognitive strategy (Manly et al., 2004; Rochester et al., 2007) although recent studies suggest that auditory-motor entrainment may be compromised in PD (Praamstra and Pope, 2007; Grahn and Brett, 2009; te Woerd et al., 2014, 2015). In healthy controls, auditory-motor entrainment (Thaut et al., 2014) relies on diverse brain areas such as the auditory cortex, the inferior parietal lobule, and frontal areas such as the supplementary motor area (SMA) and premotor cortex (PMC) (Todd and Lee, 2015). Interestingly, those regions appear to be unaffected by PD pathophysiology. Therefore, it may be possible to use NMT methodology to strengthen the aforementioned networks as a compensatory mechanism to improve motor function in PD.

We know that (1) auditory rhythm very rapidly creates stable internal reference intervals to guide the timing of motor responses and that (2) the dominant synchronization strategy is based on frequency entrainment. Entrainment of distant brain regions most likely relies on synchronization at specific frequencies that can be recorded via whole brain neuroimaging modalities such as magnetoencephalography (MEG).

No study to date reports the neurophysiological evidence of auditory-motor frequency entrainment after a NMT intervention in the Parkinson's community. We wanted to share a neuroimaging case-proof of concept series of visible changes that suggest potential mechanisms and provide further education on the clinical applications of musical interventions for motor impairments. We hypothesized that NMT-related motor improvements in PD are due to entrainment-related coupling between auditory and motor activity resulting from an increased functional connectivity between the auditory cortex and the motor cortex.

## Methods

Three right-handed PD participants were recruited from the University of Colorado Hospital Movement Disorders clinic and signed informed consents to participate in the study approved by the Colorado Multiple Institution Review Board. Inclusion criteria included a diagnosis of probable PD according to the UK Brain Bank Criteria (Hughes et al., 1992). All study visits were performed in the PD subjects' best dopaminergic “On” state. Participants' characteristics can be found in the Supplementary Table 1.

### Neurologic Music Therapy Intervention

Fifteen sessions of somatosensory-related NMT techniques were administered 3 times per week for 5 consecutive weeks by one of the NMT-certified music therapists from Rehabilitative Rhythms, Aurora, CO. Each session consisted on bimanual exercises using a keyboard, castanets and miscellaneous objects to strengthen fine motor muscles. Each finger movement was cued by either a metronome or beats produced by the therapist playing a musical instrument.

### Motor Assessments

Fine motor-related changes were assessed and quantified before and after NMT within 2 days from the first and last NMT session. We chose three different assessments to cover overall motor function, fine motor coordination and bradykinesia as well as PD-specific dexterity in order to capture the expected benefits on those symptoms:

1. The Unified Parkinson's Disease Rating Scale (UPDRS, Fahn et al., 1987) Section 3 (Motor Examination). The UPDRS is an overall marker for Parkinson's disease progression, symptoms severity and a validated measure of treatment-related benefits.
2. The Grooved Pegboard Test, which is a manipulative dexterity test consisting of 25 holes with randomly positioned slots (Trites, 1989) commonly used as a test of fine motor performance (Bryden and Roy, 2005) and general slowing due to medication or disease progression. In PD, the GPT has also been used extensively as a motor outcome of clinical trials (Haas et al., 2006).
3. The Finger-Thumb Opposition Task is one item from the Neurological Evaluation Scale (Buchanan and Heinrichs, 1989), which assesses different sensory and motor functions. Participants were asked to perform bilateral finger-thumb appositions during a 2-min lag to quantify fine motor coordination and bradykinesia.

### Magnetoencephalography Data Acquisition, Preprocessing, and Coregistration With Structural MRI

Neuromagnetic data was acquired using a Magnes 3,600 whole head MEG device with an array of 248 sensors (4D Neuroimaging, San Diego, CA) in a magnetically shielded room (ETS-Lindgren, Cedar Park, TX, USA). Participants were asked to tap with their right index finger with an acoustic burst stimuli (30 ms duration at 2,000 Hz, intensity of 70 dB above subjective threshold) delivered in their right ear every second. A quick practice session was performed prior to the MEG recording session. A total of 6 sequences of 30 s separated by a 5-s rest period were presented. Data was acquired continuously at 678 Hz with an acquisition bandwidth of 0.1–200 Hz. Scalp shape and location was determined with a 3-D digitizer to allow for comparison across subjects in a common coordinate system and for co-localization with an averaged MRI brain atlas. Data was divided into 800 ms epochs. Preprocessing included 3–70 Hz band pass filtering, noise reduction, and rejection of epochs with significant artifact. Independent component analysis was used to remove eye blink and other common artifacts (Jung et al., 2000). Epochs were baseline corrected using 800 ms baseline epochs that were extracted within the inter-block trials rest periods to prevent contamination with extended motor signals. A mean of 229 ± 18 (before NMT) and 215 ± 49 (after) epochs were subjected to further analysis. Participants' response occurred on average between 31.6 ms before (anticipatory response) and 123.7 ms after the stimulus. Stimulus-locked spectral analysis was performed over a 0–400 ms time period (0 being tone onset) in order to fully capture entrainment-related coupling between auditory and motor activity. Each participant's MEG data were co-registered with structural T1-weighted magnetic resonance imaging (MRI, Supplementary Materials) data prior to source space analyses using common landmarks from the MEG digitization procedure and MRI scan data via SPM12 software (Statistical Parametric Mapping; Wellcome Department of Cognitive Neurology, London, UK) (Friston, 2007).

### MEG Source Analysis and Source Space Statistics

Source analysis was performed in Matlab (2010b; MathWorks, Inc., Natick, MA, USA) using the SPM12 toolbox. Following co-registration of the MEG fiducials with each participant's MRI, leadfields were computed using a single shell volume conductor model. Source localization was then performed using a cortically constrained group minimum norm inversion with multiple sparse priors (Litvak et al., 2011), on all subjects' data pooled together from the three participants, which resulted in common source space images across subjects. The cortical surface used was a standard MNI space surface with 20,484 vertices supplied within SPM12. Source analysis was performed on the 15–80 Hz passband between 0 and 400 ms. Source space images were submitted to GLM-based statistical analysis using a one-sample *t*-test across all subjects to confirm the involvement of auditory and motor cortices as well to extract peak MNI coordinates in areas that survived multiple comparison correction, using a family wise error (FWE) of *p* < 0.05.

### Source Waveforms, Spectral Analyses, and Functional Connectivity

Regional time-courses were created via source-space projection (Tesche et al., 1995) from dipoles within both regions of interest: left auditory and primary motor cortices. Using the peak MNI coordinates obtained in the previous step (left auditory: −52 −35 15, left motor: −7 −25 73), the lead field and its pseudoinverse were computed and the following current source waveform (Ross et al., 2000) was created. Time-frequency transformations were then obtained using a Morlet wavelet decomposition with wave number linearly increasing from 3 to 12 across the frequency range of 15–80 Hz, on the epochs from 0 to 400 ms. Evoked power relative to the rest period baseline was calculated and averaged across subjects. In order to evaluate directional functional connectivity between our regions of interest in the frequency domain, we computed frequency domain coherence using the Fieldtrip connectivity analysis functions (Oostenveld et al., 2011), which first involved an autoregressive model fit to the data using the bsmart matlab toolbox (Cui et al., 2008). For these analyses, we downsampled the data to 250 Hz for better model order estimation and submit the data to detrenting, differencing, and pre-whitening. Then, we estimated the model order to be 16 using ARfit toolbox for Matlab (Schneider and Neumaier, 2001).

## Results and Discussion

Five weeks of NMT had beneficial effects on fine motor function in our cohort of three patients with PD (Supplementary Figure 1). PD-specific overall motor assessments showed clinically significant improvements after NMT (A). Score improvements were more mitigated for the Grooved Pegboard test, for which the dominant hand from two out of three subjects exhibited higher proficiency at picking and placing the pegs into their designated holes (B). Lastly, finger-thumb opposition test scores were greatly improved after NMT sessions, here again for two out of three participants, regardless of the hand tested (C). While fine motor assessments did not show consistent improvements among all participants, each one benefited in one or more areas of fine motor function, including the dominant hand or both hands. Interestingly, we found that finger tapping before the cue (anticipatory response) during the MEG recording occurred 73.72% before NMT whereas after NMT 90.31% of the trials were anticipatory, suggesting that NMT may enhance anticipatory motor behavior. These results are in agreement with other behavioral interventions in the PD community (Alves Da Rocha et al., 2015). In addition, this extends the benefits of NMT from gross motor to fine motor skills.

**Figure 1:**
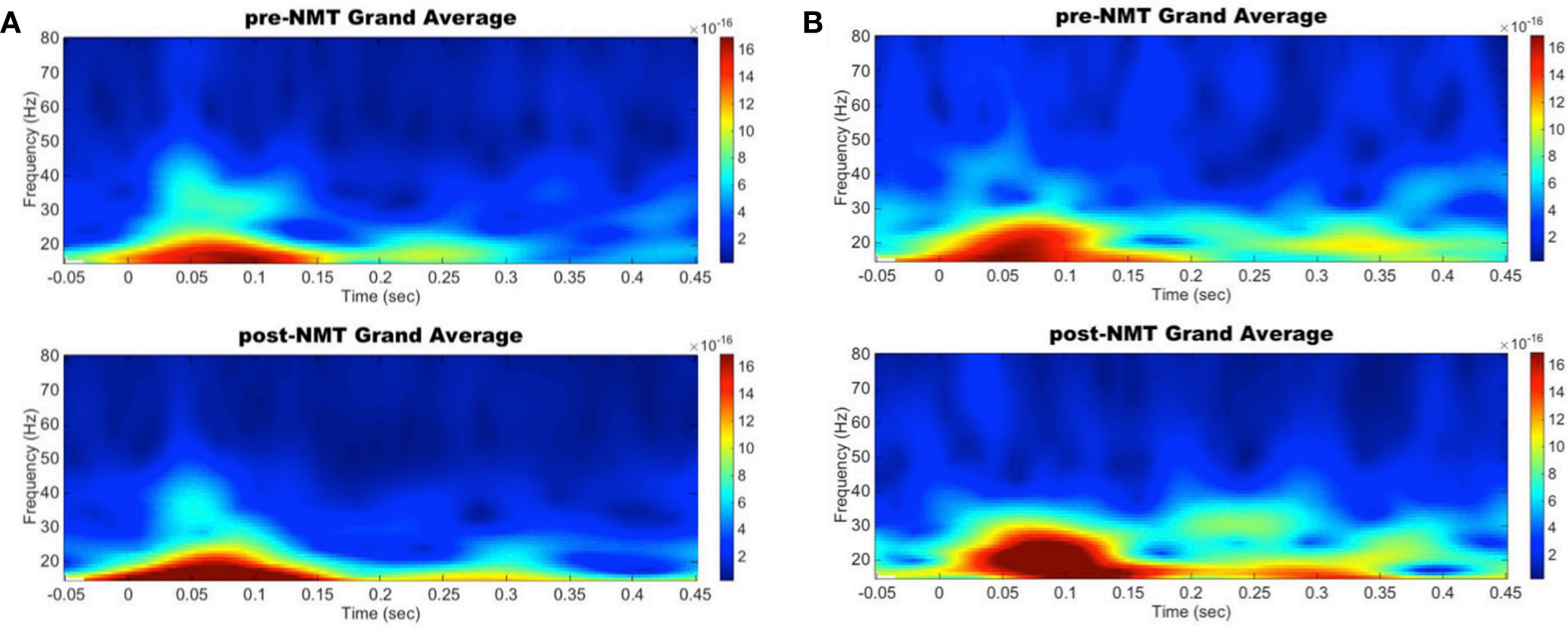
Left primary auditory and motor areas evoked power time-frequency results. Grand average evoked power in the beta range increased simultaneously in the auditory **(A)** and motor **(B)** cortices between pre-NMT (top) and post-NMT (bottom) during a cued right hand finger tapping.

Spectral analysis in the primary motor and auditory cortices during a cued finger tapping task showed a possible coinciding increase in evoked power in the beta-range suggesting an activity coupling in those two areas most likely due to their simultaneous activation (Figure 1). While this case report lacks a control group and statistical analysis, we demonstrate here NMT-related changes in cortical beta activity, an oscillation that is definitely challenged in PD. Other interventions, especially physical therapies, have been shown to modify sensorimotor alpha and beta rhythms (Mierau et al., 2009). Our results therefore suggest that musical interventions may also hold potential to influence cortical activity. Regardless of the specific pathways underlying this phenomenon, it appears that information related to the beat is simultaneously perceived by the auditory and the motor cortices, both regions we postulated would be more highly connected after NMT training.

**Figure 2:**
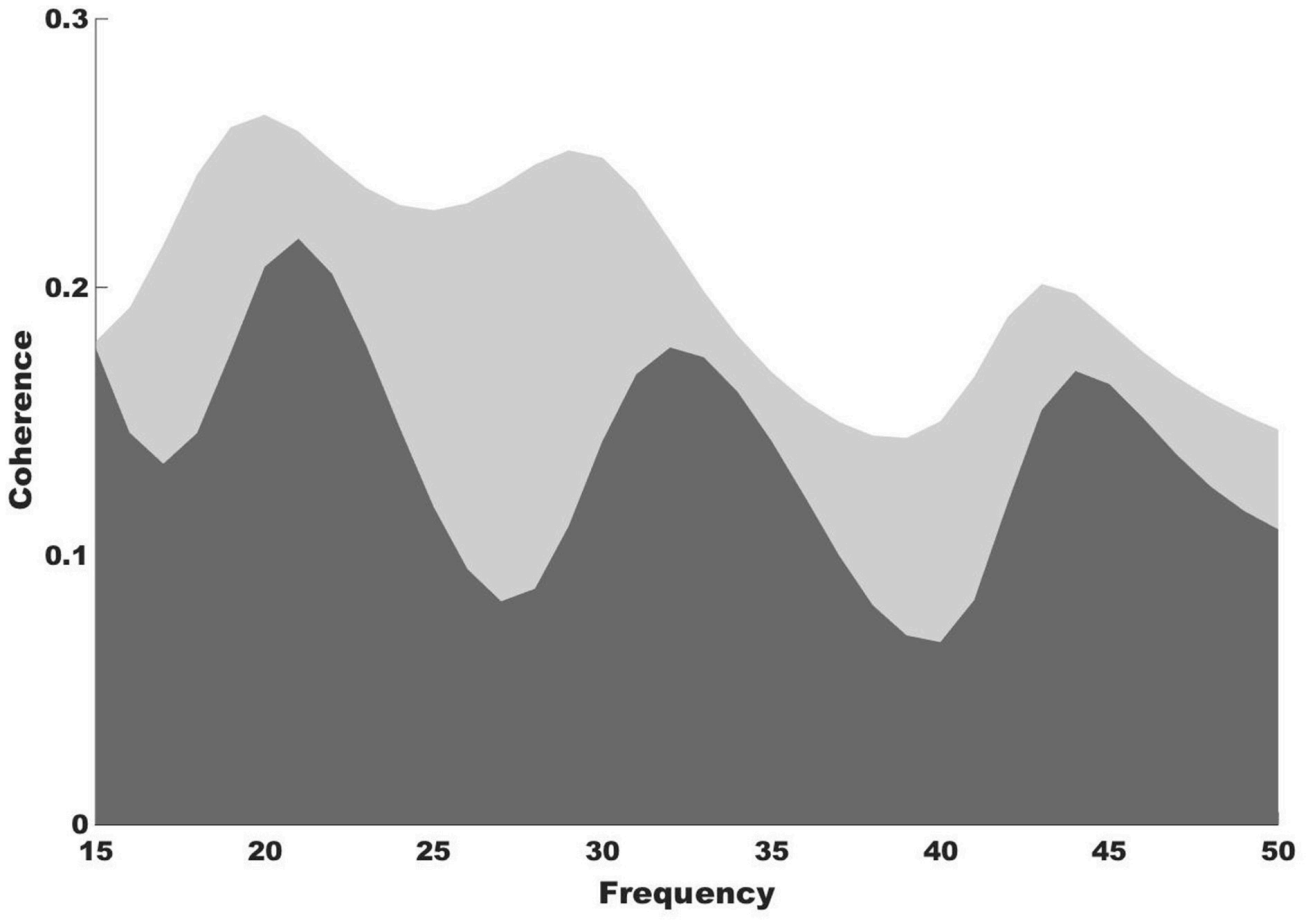
Coherence results. Coherence spectra show increased functional connectivity between the auditory and motor cortices after (light gray shade) compared to before NMT (dark gray shade).

Stronger functional connectivity between the auditory and motor cortices was observed after NMT (Figure 2). It is highly possible that the NMT-related increased connectivity between the auditory and motor cortices explains the simultaneous beta power increase in auditory and motor areas. Increased auditory-motor functional connectivity is indeed observed during synchronization to rhythm (Chen et al., 2006), which suggests a relationship between brain connectivity and rhythmic entrainment. While beat perception has been attributed to the putamen, the outermost portion of the BG, it is possible that NMT uses alternative relays to drive impaired areas via intact ones in PD. The use of brain imaging techniques with subcortical resolution will help investigating this idea.

## Conclusion

This case study provides very preliminary evidence that NMT-based motor rehabilitation may enhance cortical activation in the auditory and motor areas in a synergic manner. Our connectivity findings and the existing literature both suggest that auditory-motor connections may be improved and strengthened by training, even in the PD population. With a lack of both control subjects and control conditions, future controlled trials are warranted to further explore the effects of NMT therapy in those vulnerable patients, especially looking at symptom-specific groups given the heterogeneity of the motor symptoms found in patients with PD.

## Author Contributions

IB and MT: conceived and designed the experiments; IB: performed the experiments; IB and WD: analyzed the data; IB wrote the paper; IB, WD, MT, and BK: reviewed and revised the manuscript and approved the final manuscript as submitted.

### Conflict of Interest Statement

The authors declare that the research was conducted in the absence of any commercial or financial relationships that could be construed as a potential conflict of interest.

## Supplementary Material

Supplementary Figure 1 Fine motor assessments scores for both dominant and non-dominant hands before (pre) and after (post) a 5-week session of Neurologic Music Therapy. **(A)** Overall motor score, section 3 of the UPDRS; **(B)** Time to complete the pegboard, as part of the GPT (Grooved Pegboard Test); **(C)** Number of finger-to-thumb oppositions, as part of the NES (Neurological Evaluation Scale).

Supplementary Table 1 Participants' demographics and baseline characteristics. UPDRS, Unified Parkinson's disease rating scale, an overall marker for Parkinson's disease progression and symptoms severity.
