# Supplementary figures and images for: Preliminary Neurophysiological Evidence of Altered Cortical Activity and Connectivity With Neurologic Music Therapy in Parkinson's Disease
Source: Front Neurosci. 2019 Feb 19;13:105. doi: 10.3389/fnins.2019.00105 (PMC6390231; doi:10.3389/fnins.2019.00105)

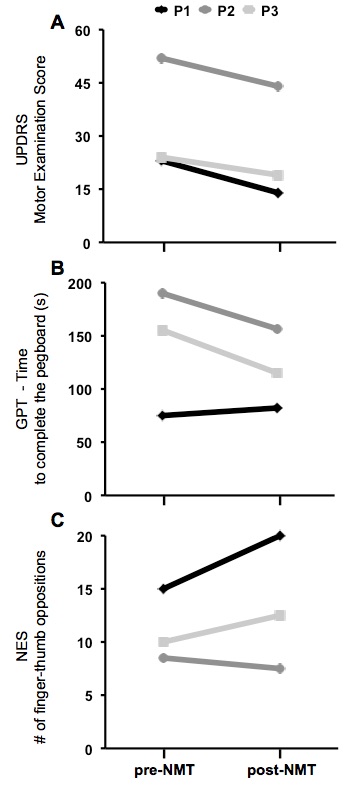

Supplement: Supplementary Figure 1 — Fine motor assessments scores for both dominant and non-dominant hands before (pre) and after (post) a 5-week session of Neurologic Music Therapy. (A) Overall motor score, section 3 of the UPDRS; (B) Time to complete the pegboard, as part of the GPT (Grooved Pegboard Test); (C) Number of finger-to-thumb oppositions, as part of the NES (Neurological Evaluation Scale). [file Image_1.JPEG]
