# Supplementary material for: Preliminary Neurophysiological Evidence of Altered Cortical Activity and Connectivity With Neurologic Music Therapy in Parkinson's Disease
Source: Front Neurosci. 2019 Feb 19;13:105. doi: 10.3389/fnins.2019.00105 (PMC6390231; doi:10.3389/fnins.2019.00105)
Supplement: Supplementary Table 1 — Participants' demographics and baseline characteristics. UPDRS, Unified Parkinson's disease rating scale, an overall marker for Parkinson's disease progression and symptoms severity. [file Table_1.docx]

|  | **Subject #1** | **Subject #2** | **Subject #3** |
| --- | --- | --- | --- |
| Age | 62 | 70 | 72 |
| Male/Female | Female | Male | Female |
| Year of education | 15 | 14 | 16 |
| Hoehn and Yahr Stage | 2.5 | 3 | 2 |
| UPDRS Total | 22 | 74 | 34 |
| UPDRS Part I: Mentation, Behavior, and Mood | 1 | 4 | 2 |
| UPDRS Part II: Activities of Daily Living | 5 | 18 | 12 |
| UPDRS Part III: Motor | 23 | 52 | 24 |
| UPDRS Part IV: Complications of Therapy | 2 | 0 | 1 |
| Levodopa equivalent dosage | 100 | 650 | 160 |

**Supplementary table. Participants’ demographics and baseline characteristics**. UPDRS: Unified Parkinson’s disease rating scale, an overall marker for Parkinson’s disease progression and symptoms severity.
