# Supplementary material for: Preliminary Neurophysiological Evidence of Altered Cortical Activity and Connectivity With Neurologic Music Therapy in Parkinson's Disease
Source: Front Neurosci. 2019 Feb 19;13:105. doi: 10.3389/fnins.2019.00105 (PMC6390231; doi:10.3389/fnins.2019.00105)
Supplement: Supplementary file 3 [file Table_2.docx]

*Magnetic Resonance Imaging*

MRI scans were acquired at the Brain Imaging Center (University of Colorado Denver, Department of Psychiatry) using a Siemens Skyra 3.0T wide bore whole body magnet equipped with high performance gradient coils (maximum gradient amplitude of 45 mT/m and maximum slew rate of 200 T/m/s), a head volume RF coil and a 12-channel phased-array head/neck coil. A T1- weighted sequence was acquired for tissue segmentation using a 3D MPRAGE sequence (matrix 256 x 256, TR/TE/TI/FLIP of 2300/2.24/900/8; 1mm square voxels), resulting in 192 1mm thick axial slices.
